# Supplementary material for: Gait and dynamic pedobarographic analyses in hallux rigidus patients treated with Keller’s arthroplasty, arthrodesis or cheilectomy 22 years after surgery
Source: PeerJ. 2023 Nov 20;11:e16296. doi: 10.7717/peerj.16296 (PMC10666645; doi:10.7717/peerj.16296)
Supplement: Supplemental Information 1 [file peerj-11-16296-s001.docx]

*Table 1: Patient demographics.^a^*

|  | Keller’s arthroplasty | Arthrodesis | Cheilectomy | Healthy controls |
| --- | --- | --- | --- | --- |
| Number of participants (number of feet) | 12 (14) | 8 (10) | 7 (9) | 13 (26) |
| Male / Female | 9 / 3 | 3 / 5 | 4 / 3 | 6 / 7 |
| Left feet / Right feet | 8 / 6 | 4 / 6 | 5 / 4 | 13 / 13 |
| Age at Surgery (years) | 54.0 (48.5-58.3) | 48.5 (43.5-54.0) | 53.0 (50.0-56.0) | - |
| Age at follow-up (years) | 75 (71-78) | 71 (65-74) | 70 (68-84) | 68 (62-73) |
| Follow-up (years) | 22 (20-22.8) | 22 (19.5-26.3) | 22 (19-25) | - |
| Height (m) | 1.72 (1.64-1.73) | 1.67 (1.51-1.74) | 1.72 (1.64-1.79) | 1.75 (1.72-1.79) |
| Body mass (kg) | 83.0 (67.0-86.0) | 81.5 (65.0-93.0) | 80.0 (74.0-87.0) | 76.0 (70.0-89.5) |
| BMI (kg/m^2^) | 27.8 (24.4-30.7) | 28.7 (26.8-37.8) | 28.4 (26.5-29.1) | 25.6 (23.2-27.5) |
| HR grade before surgery | Gr I: 2  Gr II: 8  Gr III: 3  N/A: 1 | Gr I:1  Gr II: 5  Gr III: 3  N/A: 1 | Gr I: 5  Gr II: 2  Gr III: 0  N/A: 2 | - |

*^a^Median and interquartile range are presented in parentheses*

**No statistically significant differences were detected between the groups P≥0.05*

*BMI,* Body mass index; *N/A, no preoperative radiographic results were available.*
